# Superparamagnetic Iron Oxide Nanoparticles-Complexed Cationic Amylose for In Vivo Magnetic Resonance Imaging Tracking of Transplanted Stem Cells in Stroke

**DOI:** 10.3390/nano7050107

**Published:** 2017-05-10

**Authors:** Bing-Ling Lin, Jun-Zhao Zhang, Lie-Jing Lu, Jia-Ji Mao, Ming-Hui Cao, Xu-Hong Mao, Fang Zhang, Xiao-Hui Duan, Chu-Shan Zheng, Li-Ming Zhang, Jun Shen

**Affiliations:** 1 Department of Radiology, Sun Yat-Sen Memorial Hospital, Sun Yat-Sen University, Guangzhou 510120, China; linbling@mail2.sysu.edu.cn (B.-L.L.); luliejingsysu@163.com (L.-J.L.); canterburybells@126.com (J.-J.M.); caominghui1019@163.com (M.-H.C.); xinxin110007@126.com (F.Z.); duanxiaohui-128@163.com (X.-H.D.); zhengchushan0311@126.com (C.-S.Z.); 2 Department of Polymer and Materials Science, School of Chemistry, Sun Yat-Sen University, Guangzhou 510275, Guangdong, China; zhjunzh3@mail2.sysu.edu.cn; 3 School of Materials Science and Engineering, Sun Yat-Sen University, Guangzhou 510275, China; m13929581729_1@163.com; 4 Key Laboratory for Polymeric Composite and Functional Materials of Ministry of Education, Guangdong Provincial Key Laboratory for High Performance Polymer-Based Composites, Key Laboratory of Designed Synthesis and Application of Polymer Material, Sun Yat-Sen University, Guangzhou 510275, China

**Keywords:** superparamagnetic iron oxide nanoparticles, magnetic resonance imaging, amylose, ischemic stroke, mesenchymal stem cells, green fluorescence protein, biodegradation

## Abstract

Cell-based therapy with mesenchymal stem cells (MSCs) is a promising strategy for acute ischemic stroke. In vivo tracking of therapeutic stem cells with magnetic resonance imaging (MRI) is imperative for better understanding cellular survival and migrational dynamics over time. In this study, we develop a novel biocompatible nanocomplex (ASP-SPIONs) based on cationic amylose, by introducing spermine and the image label, ultrasmall superparamagnetic iron oxide nanoparticles (SPIONs), to label MSCs. The capacity, efficiency, and cytotoxicity of the nanocomplex in transferring SPIONs into green fluorescence protein-modified MSCs were tested; and the performance of in vivo MRI tracking of the transplanted cells in acute ischemic stroke was determined. The results demonstrated that the new class of SPIONs-complexed nanoparticles based on biodegradable amylose can serve as a highly effective and safe carrier to transfer magnetic label into stem cells. A reliable tracking of transplanted stem cells in stroke was achieved by MRI up to 6 weeks, with the desirable therapeutic benefit of stem cells on stroke retained. With the advantages of a relatively low SPIONs concentration and a short labeling period, the biocompatible complex of cationic amylose with SPIONs is highly translatable for clinical application. It holds great promise in efficient, rapid, and safe labeling of stem cells for subsequent cellular MRI tracking in regenerative medicine.

## 1. Introduction

Recently, stem cell transplantation has emerged as a promising therapeutic strategy for numerous human diseases, including ischemic stroke, owing to their inherent capacity of self-renewing, homing, and multi-lineage differentiating [1,2]. The use of mesenchymal stem cells (MSCs) has many advantages, such as the simplicity of isolation and expansion, and biological properties of secreting many bioactive immunoregulatory and pro-regenerative macromolecules [3], and they have been widely-used in regenerative therapies [4]. Previous studies have proven the potential of MSCs to regenerate neural cells after being grafted into the central nervous system (CNS), thus, MSCs have been investigated as an alternative candidate for treatment of CNS disorders, including stroke [5]. Cellular therapy with the use of MSCs could obviate the need for acquiring autologous human neural stem cells for the nervous system, which is an admittedly difficult procedure. In contrast, MSCs can be isolated directly from a small volume of bone marrow or adipose tissue under local anesthesia, and can be easily expanded in vitro [6].

Imaging of transplanted stem cells in a non-invasive manner is essential because it can provide insight into cellular proliferation dynamics, biodistribution, migrational dynamics, differentiation processes, and participation in tissue repair [7]. Magnetic resonance imaging (MRI) is considered as the most suitable non-invasive method for in vivo stem cell tracking, due to its inherent non-invasiveness, absent threat of exposure to ionizing radiation, deep tissue penetration, wide clinical applicability, superior spatial resolution, and its provision of detailed information about the host tissue status. So far, MRI has been initially applied for the successful tracking of transplanted stem cells in clinical settings [8]. For cellular MRI, direct cell labeling with superparamagnetic iron oxide nanoparticles (SPIONs) became the most commonly used strategy in terms of the biocompatibility, low toxicity, and high sensitivity of SPIONs. However, the unmodified SPIONs could not be efficiently up-taken by non-phagocytic stem cells per se [9,10]. To facilitate cellular uptake, SPIONs were usually modified with a hydrophilic, biocompatible polymer coating [11]. The common coatings were based on transfection agents (TAs) like poly-l-lysine (PLL), liposome, and sulfate protamine [12]. However, cell labeling with TAs has saturation effects, requiring a high concentration of TAs and a long incubation period, which would pose a negative effect to cell biological behaviors after their transplantation [13].

Biopolymers are made of simple biological compounds produced by living organisms in the nature, such as polysaccharides, proteins, and nucleic acids. Generally, they are biodegradable, non-toxic, environmentally friendly, and thus have excellent functional properties and sustainable development advantages [14,15]. Among these renewable materials, naturally-occurring polysaccharides have attracted attention in the biomaterial field due to such advantages as outstanding non-toxicity, biocompatibility, biodegradability, capacity to associate with a broad range of molecules via chemical or physical interactions, and large-scale production at low costs [10,11,12,13,14,15,16]. They are composed of recurring units of monosaccharide or disaccharides bound together by glycosidic linkages, and can form either linear or branched polymers in structure. To date, several classes of polysaccharides (such as starch, agarose, alginate, chitosan, dextran, pullulan, and heparin) have been used to coat SPIONs [17]. Amylose, found in starch granules with amylopectin, which has been approved by the U.S. Food and Drug Administration (FDA), is a naturally abundant, inherently neutral, helical polysaccharide [18]. Amylose has such versatile traits like biocompatibility, biodegradability, capacity to be easily modified and improved transfection efficiency [19]. Further, it possesses smaller steric hindrance, better flexibility, and higher tensile strength in the physical properties compared with starch [20]. The amylose itself and its derivatives have been widely applied in biomedical field, especially as delivery carriers for drug [21,22,23] and non-viral gene vectors [24,25]. Ideally, amylose could be degraded into glucoses by the glycoside hydrolase existed in lysosomes and eventually reduced to carbon dioxide and water [9]. However, whether amylose can be used to modify SPIONs for cellular MRI has not been well tested. Biogenic amines (BAs), which are also widespread in the nature, are organic bases endowed with biological activity. Polysaccharides functionalized with either cationic amino or ammonium has been used in molecular biology [26]. Spermine, one of natural polycationic biogenic amines required for growth and differentiation in all eukaryotic cells [27], has been used in cationization of polymers to act as a gene vector [28]. Spermine-modified amylose (ASP) was originally applied as a gene carrier [29]. However, to the best of our knowledge, whether ASP can be used as a biomaterial to label cells for MRI tracking remains unknown.

In this work, we developed a novel type of nanometer-sized SPIONs-complexed amylose nanoparticles cationized with spermine (ASP-SPIONs) to label transgenic green fluorescent protein (GFP)-MSCs for in vivo MRI tracking. To this end, the naturally neutral, hydrophobic amylose from starch was modified with spermine grafting to be cationic and hydrophilic, which facilitated the cellular uptake after complexed with SPIONs. After MSCs were labeled with ASP-SPIONs, the labeling efficacy and safety were determined in vitro. The distribution and migration of the labeled cells after transplantation were detected with the use of in vivo MRI in an experimental rat model of acute focal ischaemic stroke over a six-week-long period of follow-up. The aim of this study was to explore the potential of ASP-SPIONs as a novel biocompatible polysaccharide-based biomaterial to label stem cells magnetically, and to meet the requirement of subsequent in vivo MRI tracking of therapeutic cells in regenerative medicine.

## 2. Results

### 2.1. Synthesis and Characterization of Nanoparticles

#### 2.1.1. Synthesis and Characterization of ASP

**Figure 1:**
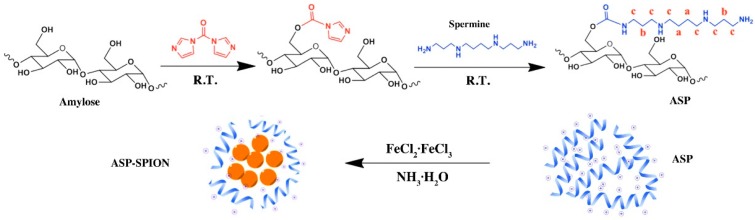
Schematic diagram of the synthesis of cationic superparamagnetic iron oxide nanoparticles (SPIONs)-complexed spermine-modified amylose (ASP), which is denoted as ASP-SPIONs. R.T.: room temperature.

The hydrophobic amylose was functionalized with spermine to synthesize cationic ASP, by using the CDI activation method [30]. The rich hydroxyl groups on the glucose chain of amylose react with CDI to give the carbonylimidazole residues, which react further with spermine to form the carbamate structures (Figure 1). The degree of substitution (DS) of ASP was 0.17, which could be calculated by N content via elementary analysis. As shown in Figure S1, FT-IR spectra demonstrated that the spectrum of ASP showed two new peaks compared with that of primary amylose. Peak at 1709 cm^−1^ could be assigned to the C=O vibration of carbamate structures while peak at 1265 cm^−1^ could be ascribed to C–N vibrations of aliphatic amines. The results indicate that spermine was grafted to amylose backbone with carbamate linkages. Furthermore, as shown in Figure S2, the proton peaks at 1.45, 1.63, and 2.62 ppm of ^1^H NMR spectra confirmed that the oligoamine residues were conjugated with amylose [31].

#### 2.1.2. Synthesis and Characterization of ASP-SPIONs

ASP-SPIONs were fabricated via one-pot synthesis in a solution phase at high temperature [32]. This method did not require any extra surface modification (Figure 1). Fourier transform infrared (FTIR) spectra confirmed the presence of cationic amylose, as demonstrated by a comparison between SPIONs and ASP-SPIONs (Figure S3). The absorption peaks at 580 cm^−1^ belonged to the typical characteristic Fe-O vibration band in SPIONs. ASP-SPIONs showed characteristic absorption bands for C=O at 1709 cm^−1^, C–N at 1265 cm^−1^ and Fe–O at 580 cm^−1^. 

For the resultant ASP-SPIONs, their thermogravimetric (TG) curve, X-ray diffraction (XRD) pattern, and magnetization curve were assessed and compared with those of SPIONs. Transmission electron microscopy (TEM) images were also obtained to characterize ASP-SPIONs. As demonstrated in Figure S4, SPIONs had a mass loss of 3.07%, while ASP-SPIONs had a greater mass loss of 64.10%. XRD patterns of ASP-SPIONs and SPIONs are shown in Figure S5. Both ASP-SPIONs and SPIONs showed the characteristic diffraction peaks, which were marked respectively by their indices (220), (311), (400), (422), (511), and (440) [33].

As shown in Figure S6, the saturation magnetization was found to be 68.7 emu/g for SPIONs and 22.4 emu/g for ASP-SPIONs. In addition, the magnetization curves of both ASP-SPIONs and SPIONs exhibit nearly no remanence. These results indicate that SPIONs with a modification of ASP remained superparamagnetic, which is suitable for MRI [33].

**Figure 2:**
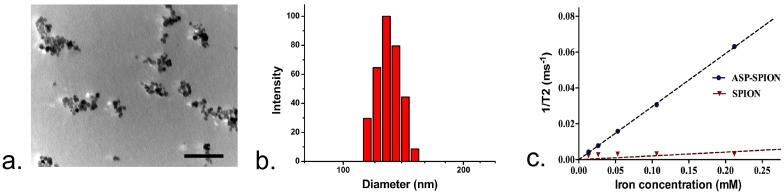
Characterizations of ASP-SPIONs: (**a**) Transmission electronic microscopy (TEM) image demonstrates the shape of ASP-SPIONs and the modification of SPIONs by ASP, resulting in relatively small amounts of aggregates; scale bar: 100 nm. (**b**) The hydrodynamic diameter distribution of ASP-SPIONs. (**c**) The r2 relaxitivity of ASP-SPIONs compared with native SPIONs.

TEM revealed the shape of ASP-SPIONs was amorphous, but not typically spherical (Figure 2a). This might be assumed to be due to the hydrophilic nature of the nanoparticles, as amylose was modified by only one molecule of spermine. The particle size and zeta potential of ASP-SPIONs measured by dynamic light scattering (DLS) were 128.2 ± 1.8 nm and 21.59 ± 1.92 mV, respectively (Figure 2b), while the size of the individual SPIONs was 14.6 ± 1.4 nm. The iron content of ASP-SPIONs was 250 µg/mL. The r2 relaxivity of ASP-SPIONs was 296.2 mM^−1^·S^−1^, while the r2 relaxivity of native SPIONs was 21.1 mM^−1^·S^−1^ (Figure 2c). 

#### 2.1.3. Aqueous Dispersibility

The introduction of amino groups turns ASP to be highly soluble. As shown in Figure S7, the aqueous dispersions of ASP-SPIONs could maintain macroscopically homogeneous for 1 month, while the aggregation and sedimentation of native SPIONs would occur in water. The good aqueous dispersbility of ASP-SPIONs might be attributed to hydrophilic ASP. After α-amylase assay, the SPIONs in the complexes aggregated and precipitated due to the destruction of the amylose backbone by α-amylase.

### 2.2. Optimal Labeling Condition of ASP-SPIONs

**Figure 3:**
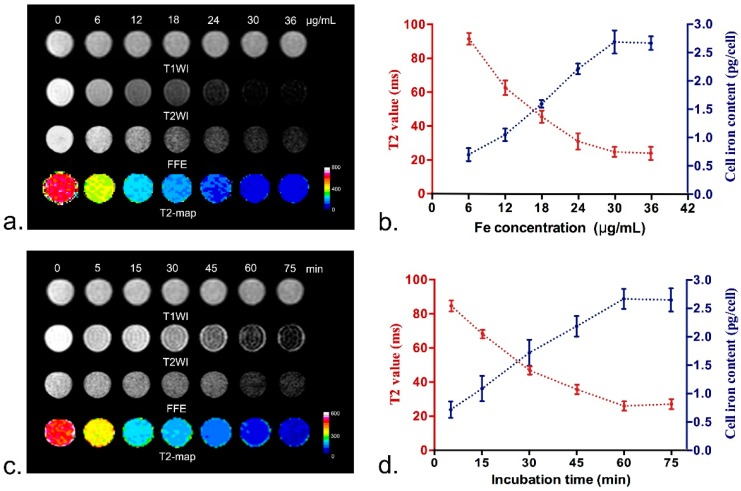
Optimal labeling conditions of ASP-SPIONs. MR images (**a**,**c**) and graphs (**b**,**d**) show that with the increase in the concentration (**a**,**b**) or incubation time (**c**,**d**). T2 values and iron content per cell of labeled cells exhibit a non-linear change , as quantified by atomic absorption spectrometry (AAS), and T2-map (*n* = 6 for ASS and *n* = 3 for 2-values). T2-map was pseudocoloured with a look-up table using ImageJ software. This change is consistent with the signal intensity changes on T1-(T1WI), T2-(T2WI) and T2*-weighted images (T2*WI or FFE). The optimal labeling conditions for ASP-SPIONs were an iron concentration of 30.0 μg/mL and an incubation time of 1 h, which was determined by the highest iron concentration per cell and the lowest T2 values.

The optimal labeling conditions with ASP-SPIONs were assessed by in vitro MRI and atomic absorption spectrometery (AAS). As shown in Figure 3, with the increase of incubation time and iron concentration, T2 values and iron content in a single, labeled cell exhibited a non-linear change. In vitro MRI showed that cells labeled with ASP-SPIONs demonstrated remarkably decreased signal intensity on T2-weighted imaging (T2WI), T2*-weighted imaging (T2*WI), and decreased T2 values compared with unlabeled cells, when cells were labeled under the condition of 1 h incubation time and the iron concentration of 30.0 µg/mL (Figure 3a,c). AAS demonstrated that the incubation time of 1h, and nanoparticle iron concentration of 30.0 µg/mL, were the most efficient for cell labeling. Cells labeled with ASP-SPIONs had a mean iron concentration of 2.68 pg per cell (Figure 3b,d). Longer incubation time (up to 75 min), or further increase of nanoparticle iron concentration (up to 36 µg/mL), did not significantly improve the cellular uptake. Thus, the optimal cell labeling conditions were determined as an incubation time of 1 h, and the iron concentration of 30 µg/mL.

### 2.3. Intracellular Distribution of Nanoparticles

**Figure 4:**
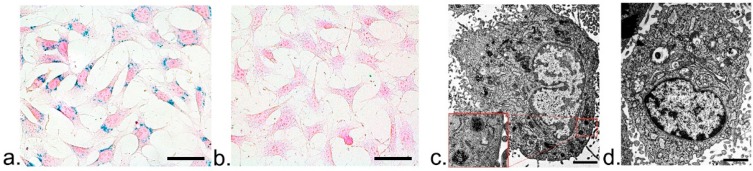
Intracellular distribution of nanoparticles. Prussian blue staining micrographs show abundant blue-stained particles in the cytoplasm around red-stained nucleus in cells labeled with ASP-SPIONs (**a**), while no such blue-stained particles are present in unlabeled cells (**b**), scale bar: 50 μm. Transmission election microscopy images show that numerous dense iron particles are present in the endosome (inserted magnified view) and cytosol of cells labeled with ASP-SPIONs (**c**), while no such iron particles are present in unlabeled cells (**d**), scale bar: 2 μm.

As shown in Figure 4, Prussian blue staining revealed that there were abundant blue-stained particles in the cytoplasm around red-stained nucleus in cells labeled with ASP-SPIONs (Figure 4a). Almost 100% of the cells were positive, which demonstrated strong intracellular accumulation of SPIONs and a high labeling efficiency. No obvious morphological changes were found after cells had been labeled with ASP-SPIONs. However, no blue staining particles could be detected in unlabeled cells (Figure 4b). TEM showed that abundant dense iron particles were located in the endosome and cytosol (Figure 4c), while no iron particles were seen in unlabeled cells (Figure 4d). 

### 2.4. Safety of Cell Labeling

**Figure 5:**
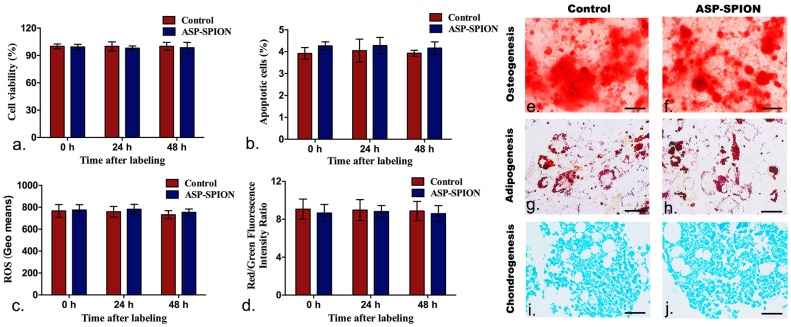
In vitro cytotoxicity assay of cell labeling. Graphs show no significant differences in the cell viability (**a**), apoptosis rate (**b**), intracellular ROS level (**c**) , and mitochondrial membrane potential (**d**) at 0, 24 and 48 h after labeling with ASP-SPIONs in comparison with control cells. Values represent means ± SD, *n* = 6, *p* > 0.05. (**e**–**j**) Presentative micrographs show that abundant calcium nodules indicating osteogenesis (**e**,**f**), red-staining oil granules indicating adipogenesis (**g**,**h**), and synthesis of proteoglycans indicating chondrogenesis (**i**,**j**) in both labeled and unlabeled mesenchymal stem cells (MSCs). Scale bar for **e**–**h**: 100 μm; scale bar for **i** and **j**: 50 μm.

Under the optimal labeling conditions, the cell viabilities remained above 90% within 48 h, and no significant difference of the apoptosis rate was detected 24 h and 48 h after labeling between cells pre-labeled with ASP-SPIONs, and unlabeled cells (Figure 5a,b, *p* > 0.05). Moreover, no increases in reactive oxygen species (ROS) production or decreases in mitochondrial transmembrane potential were observed in the labeled cells at different time points, when compared with unlabeled cells (Figure 5c,d, *p* > 0.05). Furthermore, both labeled and unlabeled MSCs showed similar osteogenic, adipogenic, and chondrogenic differentiation capacity when grown in appropriate induction culture media (Figure 5e–j). These results indicate that cell viability, apoptosis rate, intracellular ROS level, mitochondrial transmembrane potential, and multilineage differentiation capacity were not affected in MSCs when labeled with ASP-SPIONs.

### 2.5. In Vivo MRI

**Figure 6:**
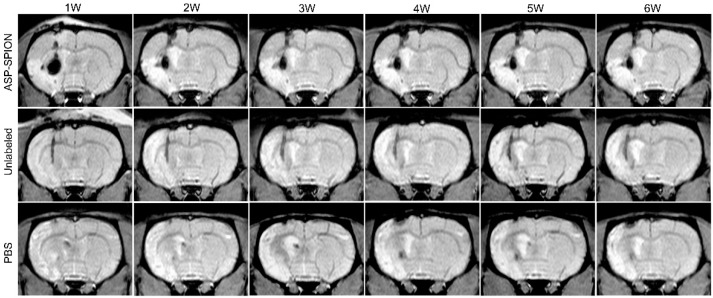
In vivo MRI tracking of the grafted MSCs. Longitudinal coronal T2*-weighed images show a persistent hypointense area within 6-week follow-up representing cell grafts in the striatum of animals that were grafted with ASP-SPIONs-labeled cells. Only a black, linear signal representing needle track is observed in the striatum in animals treated with unlabeled cells or PBS.

**Figure 7:**
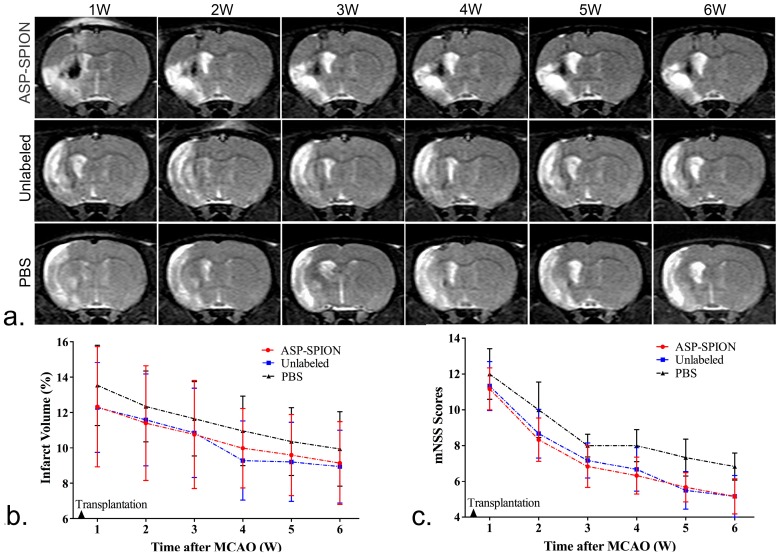
Therapeutic effects of cell transplantation. Longitudinal coronal T2-weighed images (**a**) show a less pronounced hypointense area within 6-week follow-up representing cell grafts in the striatum of animals that were grafted with ASP-SPIONs-labeled cells. Only a black, linear signal representing needle track is observed in the striatum in animals treated with unlabeled cells or PBS. Graph (**b**) shows the measured infarct volumes are similar in rats grafted with ASP-SPIONs-labeled cells and unlabeled cells, which are slightly lower than rats treated with PBS, though no significant difference was found among three groups (*p* > 0.05). Graph (**c**) shows the modified neurological severity scores (mNSS) in rats that were transplanted with ASP-SPIONs-labeled cells, unlabeled cells, and PBS. There is a similar therapeutic effect between label cells and unlabeled cells. * *p* < 0.05.

MSCs pre-labeled with ASP-SPIONs were injected into the ipsilateral striatum in rats with cerebral acute ischaemic infarct. MRI was performed to track the distribution and migration of transplanted, labeled cells. One week after transplantation, the grafted MSCs pre-labeled with ASP-SPIONs appeared as strongly hypointense areas in the left striatum on T2*WI (Figure 6) and less pronounced hypointense signal area on T2WI (Figure 7a). These hypointense signals remained consistent until 6 weeks after transplantation. However, no such developmental hypointense signal was observed in animals grafted with unlabeled cells or phosphate-buffered saline (PBS), and only a black needle tract could be observed instead.

### 2.6. Therapeutic Effects

The infarcted brain appeared as hyperintense (bright) signals on T2WI (Figure 7a). The infarct volume quantified by MRI showed a slow decline from 1 week to 6 weeks after transplantation in animals treated with ASP-SPIONs-labeled cells, unlabeled cells, and with PBS (Figure 7b). The infarct volume in those transplanted with PBS was slightly higher than in rats grafted with labeled cells or unlabeled cells, though there was no significant difference found (*p* > 0.05). Behavioral tests showed a gradual decrease of mNSS scores beginning at 1 week after transplantation in animals treated with ASP-SPIONs-labeled cells, unlabeled cells, and PBS, indicating a gradual improvement of the sensorimotor deficits (Figure 7b). The mNSS scores in rats grafted with ASP-SPIONs-labeled cells, and in those grafted with untreated cells, were significantly lower than that in rats treated with PBS at 4, 5, and 6 weeks after transplantation (*p* < 0.05), suggesting a beneficial effect of MSCs on functional recovery. No significant difference was found between ASP-SPIONs group and unlabeled group at each time point (*p* > 0.05), indicating a similar therapeutic effect between these two groups.

### 2.7. Histology

**Figure 8:**
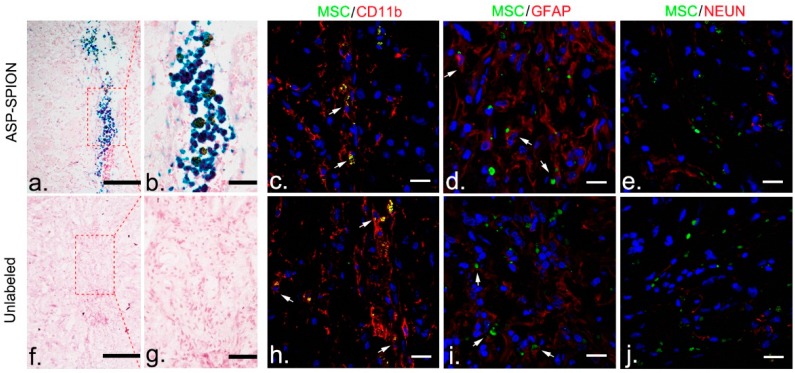
Histopathology assessment of grafted cells. At 6 weeks after transplantation, Prussian blue staining micrographs show that blue-stained cells remained in the injection site, surrounded by extracellular positive aggregates, in animals treated with ASP-SPIONs-labeled cells (**a**,**b**). No positive iron-containing cells were found in animals grafted with unlabeled cells (**f**,**g**). Scale bar for **a**,**b**,**c**,**f**: 100 μm. Fluorescence immunostaining micrographs reveal that green fluorescent protein (GFP)-MSCs remained in the injection site in animals treated with ASP-SPIONs-labeled cells or unlabeled cells. Few GFP-cells were differentiated into GFAP positive astrocyte (**d**,**i**), but no cells into NeuN positive neurons (**e**,**j**). A small number of cells were phagocytized by macrophages (**c**,**h**). Scale bar for all immunostaining micrographs: 20 μm.

Prussian blue staining demonstrated that there were numerous iron-containing cells in the injection site at 6 weeks after transplantation in animals treated with ASP-SPIONs-labeled MSCs (Figure 8a,b). Extracelluar positive particles were also found alongside the positive cells in the implantation site. No positive cells were observed in animals treated with unlabeled MSCs (Figure 8f,g). Fluorescence immunostaining showed that there were considerable surviving MSCs (GFP positive cells) in the implantation sites in animals treated with ASP-SPIONs-labeled or unlabeled cells. Few viable, grafted cells were differentiated into GFAP positive astrocytes (Figure 8d,i), while a minority of cells were double positive for GFP and CD11b (Figure 8c,h), indicating phagocytosis of grafted cells by macrophages. However, no differentiation of NeuN positive neurons was found (Figure 8e,j).

## 3. Discussion

In this study, we synthesized a novel, cationic nano-scaled SPIONs-loaded particle for cellular tracking, by introducing the spermine group to the biocompatible, biodegradable amylose. Our experimental results showed that MSCs can be successfully labeled via these cationic nanoparticles, with a high labeling efficiency at a low SPIONs concentration (30 µg/mL), and an obviously short labeling period (1 h incubation time). No noticeable detrimental effects were found in the cell viability, apoptosis, oxidative stress, mitochondrial transmembrane potential, multilineage differentiation capacity, and on their beneficial effect on stroke. Upon cell labeling, the distribution and dynamic migration of the transplanted stem cells could be clearly tracked in vivo with the use of MRI. Histology demonstrated that grafted cells could survive for up to 6 weeks after transplantation.

Since stem cells cannot take up appreciable amounts of unmodified SPIONs, various strategies have been used to elevate intracellular uptake of SPIONs. These include a specific approach with the use of targeting ligands or antibodies against cell receptors, and a non-specific approach using TAT protein, amphiphilic coatings (dendrimers, dextran, PEI, and PLL) and various cationic TAs such as liposome, protamine, and lipofectamine [11]. Modifying the magnetic particles with high affinity ligands enables SPIONs to be taken up specifically by the target cells via receptor-mediated endocytosis [34]. However, this strategy requires the availability of an internalized monoclonal antibody. Furthermore, the number of cell membrane receptors restricts the internalization efficiency. Though linking translocating peptides HIV Tat to the SPIONs surface facilitates particle internalization [35], the HIV tat peptide can cause the SPIONs accumulation in the nucleus and therefore may potentially interfere with nucleus function [36]. Most of the reported means are non-specific and depend on electrostatic interactions between cells and the nanoparticles in the presence of cationic transfection agents. The most commonly used commercialized SPIONs are dextran-coated Endorem and carboxydextran-coated Resovist. These cannot mediate sufficient cellular uptake of SPIONs, probably because of a relatively inefficient fluid phase endocytic pathway [37]. Generally, the non-specific methods require relatively high SPIONs concentrations and long incubation time for efficient labeling [38]. The label dose of SPIONs reported varied from a few µg/mL, to over 1 mg/mL, commonly from 20 µg/mL to 50 µg/mL. As the most frequently-used molecule in animal studies [10], and the choice of coating used for SPIONs in initial clinical trials [39], PLL labeling demanded long incubation periods (up to 24 h) for efficient cell labeling. Nevertheless, the prolonged incubation time and high dose of SPIONs could result in potential cell toxicity and exert adverse effects on cell survival and proliferation due to ROS, disruption of protein conformation, and distorted functioning of cell endosomes. These would become major concerns in terms of the safety of cell labeling [40]. In our study, the cationic ASP-SPIONs showed a high labeling efficiency at a low SPIONs concentration, and an obviously short labeling period, but without harmful effects. Such a convenient, safe labeling procedure, with the features of a rapid incubation time and a relative low SPIONs concentration, is greatly desired for clinical translation of stem cell labeling.

In the last decade, a wide variety of polymeric nanoparticles have been developed as robust carriers for the delivery of drugs, genes and imaging contrasts, showing high transmission efficiency, less toxic effects, and active targeting effects [41]. Among them, biopolymer-based nanoparticles are favorable for clinical application due to desirable features such as high biocompatibility, biodegradability, and low immunogenicity. These have already been used as drug delivery carriers, gene vehicles, and imaging labels of specific sites [42]. Amylose, as one of the most abundant polysaccharides in nature, with the great advantages of non-toxicity, biocompatibility, biodegradability, high stabilization, and large-scale production at low cost, is considered as an ideal candidate vector for cell magnetic labeling [26,27,28,29,30,31,32,33,34,35,36,37]. However, unmodified amylose is neutral and has low solubility, which are unfavorable for cellular uptake [43]. In this study, we modified amylose by grafting the spermine group to make it cationic and hydrophilic. The electrostatic (van der Waals) bonds between the positive charges of ASP and the negative charges on the cell surface cause membrane bending, which facilitates endocytosis and macropinocytosis of nanoparticles. Upon this, the imaging labels, SPIONs, are internalized into the cell lysosomes, and then released into cytosol, which can be confirmed by TEM and Prussian blue staining, respectively. ASP coating, similar to other coatings, mediates cellular uptake of SPIONs through endocytosis and micropinocytosis, eventually causing SPION accumulation in the lysosomes and dispersion in cytoplasm [44]. As mentioned above, spermine is a polymine involved in cellular metabolism in all eukaryotic cells and can be found in a wide variety of organisms and tissues. It is also a biomolecule and thus could preclude the potential immunogenicity introduced by the nanocomplex. On the other hand, spermine is widely applied in modifications of non-viral gene vectors. Polycations that were grafted with higher spermine content demonstrated higher transfection efficiency [32]. The introducing spermine into amylose also enables ASP-SPIONs the potential to integrate therapeutic DNA or RNA. Further, the hollow structure (owing to the helical amylose) and hydrophilicity (owing to spermine modification) allow it to encapsulate or integrate a broad range of drug and molecules, such as ligands, antibodies, enzymes, or functional RNAs. Thus, ASP-SPIONs has a high potential to integrate therapy, monitoring, targeting, and multimodality imaging labels into one entity. 

Our synthesized ASP-SPIONs had a relatively high r2 relaxivity, as 14-fold (296.2 mM^−1^·S^−1^ versus 21.1 mM^−1^·S^−1^). This was higher than that of free SPIONs in aqueous solution, which is superior to three commercially-available T_2_ contrast agent, including Resovist (180~202 mM^−1^·S^−1^), Endorem (about 120 mM^−1^·S^−1^), and Sinerem (about 65 mM^−1^·S^−1^). There are many factors that can affect the r2 relaxivity of SPIONs-loaded nanoparticles, such as the shape, size, clustering status, and volume fraction of magnetic materials inside. As ASP-SPIONs can incorporate many SPIONs in a single nanoparticle, we believe that this clustering effect of SPIONs results in the higher r2 relaxivity of ASP-SPIONs, compared with naive SPIONs. 

In vivo MRI tracking could help to reveal the long-term fate of grafted stem cells in acute ischemic stroke. In this study, we transplanted MSCs that were already genetically modified with the GFP gene. As such, it can be easy to track and identify living cells. On MRI, the grafted MSCs pre-labeled with ASP-SPIONs were detected as a persistent, localized hypo-intense signal in the injection site, up to 6 weeks after transplantation. With the use of the fluorescent GFP label, our results showed that labeled MSCs can survive for a long period of 6 weeks after transplantation. The distribution of grafted cells could be clearly detected by MRI. On the other hand, the hypointensity areas detected by MRI can be assumed to the retained SPIONs in surviving cells. Further, they can also be assumed to extracellular SPIONs aggregates derived from the release of previously endocytosed contrast agents with cell apoptosis, or phagocytosis of grafted cells in the implantation site by reactive macrophages [45]. Similar results were observed in acute myocardial infarction [46]. Thus, in vivo MRI tracking of stem cell was effective and could verify the biodistribution of cell grafts, but it may overestimate the true size of viable stem cell mass in damaged brain tissue. In our study, we did not find significant differences in the infarct volume between animals treated with the PBS, and with labeled or unlabeled MSCs. However, the functional recovery was better in animals treated with MSCs than those with PBS. This finding suggests that cell labeling with ASP-SPIONs does not affect the therapeutic effect. Stem cells can promote functional recovery by the production of an array of neurotrophins and bioactive factors, such as VEGF, insulin growth factor-1 (IGF), and nerve growth factor. As for structural recovery, a large quantity of stem cells might be needed due to massive cell death after cell implantation into the infarction. The infarct volume detected by MRI only reflects structural changes, but not functional changes. It is reasonable that the evolution of the infarct volume did not achieve a significant difference under the current transplantation paradigm (5 × 10^5^ MSCs in acute phase of cerebral infarction).

## 4. Materials and Methods

### 4.1. Animals

Eighteen adult male Sprague-Dawley (SD) rats (weighing 250–280 g), obtained from the Animal Experiment Center of Sun Yat-Sen University, were housed in a standard animal facility with food and water available ad libitum, and were used to establish acute focal ischaemic cerebral injury. All experiments were approved by the Institutional Animal Care Committee of Sun Yat-Sen University (Projection identification code: [2015]077; 3 March 2015), and all procedures complied with the guidelines for the care and use of laboratory animals, and the ethics review process of Sun Yat-Sen Memorial Hospital. 

### 4.2. Synthesis and Characterization of Nanoparticles

#### 4.2.1. Materials

Amylose (> 98%) from potato was purchased from ScienMax Inc. (Plano, TX, USA). Molar mass distribution of the purified amylose obtained, as detected by gel permeation chromatography (GPC), was: M*n* = 83.0 kDa, M_W_ = 107.6 kDa, polydispersity index (PDI) = 1.30. Spermine with purity of 97% was purchased from Alfa Aesar (Ward Hill, MA, USA). *N*,*N*’-carbonyldiimidazole (CDI) with purity of 98%, and dry DMSO, were bought from J&K Scientific Ltd. (Beijing, China). FeCl_3_·6H_2_O, FeCl_2_·4H_2_O, 25% NH_3_·H_2_O, and other reagents, were of analytical grade and used as received.

#### 4.2.2. Preparation of Cationic Amylose

The cationic amylose was synthesized by introducing spermine to the hydroxyl groups of amylose using the CDI activation method (Figure 1). Briefly, 0.30 g amylose was dissolved in 15 mL dry DMSO, and then 0.30 g CDI was added and stirred for 2 h under nitrogen atmosphere at room temperature. After that, 0.30 g spermine was added and the reaction was allowed to proceed in N_2_ at room temperature. After 24 h, the resultant reaction mixture was dialyzed against distilled water for 3 days (MWCO = 14,000), and lyophilized to obtain the spermine-functionalized amylose (ASP).

#### 4.2.3. Preparation of SPIONs-Complexed Cationic Amylose

The SPIONs-complexed ASP (ASP-SPIONs) were synthesized by the one-pot method (Figure 1). Briefly, 0.20 g ASP was dissolved in 20 mL of deionized water. 5 mL aqueous solution containing 0.20 g FeCl_3_·6H_2_O and 0.10 g FeCl_2_·4H_2_O was added, and the mixture was purged with N_2_ for 30 min. Then, 2.5 mL 25% NH_3_·H2O was added under vigorous stirring and the reaction was allowed to proceed for 1 h at 80 °C. The mixture was cooled to room temperature and dialyzed against distilled water for 2 days (MWCO = 14,000). After centrifugation, the upper aqueous dispersion of ASP-SPIONs was collected and stored at 4 °C. The black solid of ASP-SPIONs was obtained by lyophilization.

#### 4.2.4. Characterization

The FTIR spectra was carried out on a Nicolet/Nexus 670 FT-IR spectrophotometer (Thermo Nicolet, Madison, WI, USA) at a resolution of 4 cm^−1^ and frequencies ranging from 450 to 4000 cm^−1^. ^1^H NMR analysis was performed by AVANCE III 400 MHz NMR Spectrometer (BrukerBiospin, Fällanden, Switzerland) at room temperature using DMSO-d_6_ or D_2_O as the solvent. The TG analyses (TGA) were carried out on a Pyris 1 thermogravimetric analyzer (PerkinElmer, Waltham, MA, USA). For TGA, the samples (about 3 mg) were heated from room temperature to 700 °C at a heating rate of 20 °C/min in N_2_. The XRD patterns were recorded on a SmartLab X-ray diffractometer (Rigaku, Tokyo, Japan) using Cu Kα radiation. The magnetic measurements were carried out on a model MPMS XL-7 magnetometer (Quantum Design, San Diego, CA, USA).

The TEM images were obtained on a JEM-2010HR 200 kV transmission electron microscope (JEOL, Tokyo, Japan). For TEM, the sample suspension was prepared by drying a drop (5 μL, 0.5 mg/mL) of the sample solution on a copper grid coated with amorphous carbon, and then blotted with a filter paper after 1 h. After staining the samples with 10 μL of uranyl acetate solution (2 wt % in water), blotted with a piece of filter paper after 1 min, the grid was then finally dried in the air. The measurements of particle sizes and zeta potentials were performed on a ZetaPALS instrument (Brooken Haven, NY, USA). To detect the iron content, the sample were dissolved in 1 M HCl solution for thorough release and dissolution of SPIO, then analyzed at the specific Fe absorption wavelength (248.3 nm), based on a pre-established calibration curve using a polarized Zeman atomic absorption spectrometer (AAS; Hitachi Z-200; Tokyo, Japan). The r2 relaxivity of the obtained ASP-SPIONs was detected by using a 3.0 T MRI scanner (Intera; Philips Medical Systems, Best, The Netherlands) with an 8-channal sense knee coil. The measurements were performed by using single-slice multi-echo spin echo (SE) imaging for transverse relaxation time (T2) calculations to generate T2-maps. The acquisition parameters were: repetition time (TR)/echo time(TE) = 2000/20–80 ms, 4 stepped echoes, and number of acquisitions (NSA) = 1, acquisition matrix = 160 × 266, field of view (FOV) = 80 mm × 80 mm, and slice thickness = 2 mm. Dispersion stability assay was carried out by adding α-amylase (3 unit/mL) into ASP-SPIONs dispersion (1 mg/mL), and incubating in 37 °C water bath.

### 4.3. Cell Preparation

GFP-MSCs were purchased from Cyagen Biosciences (Guangzhou, China), which were derived from SD rats through bone marrow adherent method and transfected with a GFP expressing lentiviral construct. GFP-MSCs have a strong capacity to expand and constitutively express GFP. The MSCs were grown in a culture medium consisting of Dulbecco’s Modified Eagle Medium (DMEM, GiBco, NY, USA), supplemented by 10% fetal bovine serum (FBS, GiBco, NY, USA), 1% penicillin, 1% streptomycin, and 1% glutamine (Sigma, St. Louis, MO, USA) at 37 °C under a 5% CO_2_ atmosphere, and underwent routine passage with a ratio of 1:2 to 1:3. Cells between fifth and ninth passage were utilized in the following experiments.

### 4.4. Optimal Cell Labeling Conditions

To determine the optimal labeling concentration with ASP-SPIONs, MSCs were seeded in a 96-well plate at a density of 5 × 10^5^ cells/well, containing a total of 200 µL culture medium. When MSCs reached 90% confluence, the culture medium was replaced with 200 µL fresh culture medium, then nanoparticles were separately added to each well to label cells, with final iron concentrations of 6, 12, 18, 24, 30, and, 36 µg/mL, respectively. The cells were incubated for 60 min under standard culture conditions (37 °C, 5% CO_2_). To determine the optimal incubation time, 24 µL of nanoparticle was added and incubated with cells under standard culture conditions for 5, 15, 30, 45, 60, and 75 min, respectively. Untreated cells were used as controls. After labeling, the cells were thoroughly washed with PBS three times and collected after trysinization. The intracellular uptake of SPIONs was then quantified by in vitro MRI and AAS. For MRI, cells were resuspended in 200 μL 1% agarose solution and imaged after gelation. MRI was acquired by using a clinical 3.0-T system (Intera; Philips Medical Systems, Best, The Netherlands). Fast spin echo (FSE) T1WI (TR/TE = 500/15 ms; NSA = 3; Acquisition matrix = 229 × 290), T2WI (TR/TE = 2000/100 ms; NSA = 3; Acquisition matrix = 268 × 267) and fast field echo (FFE) T2*WI (TR/TE = 300/11.5 ms; Flip angel = 20°; NSA = 4; Acquisition matrix = 268 × 296) were obtained. T2 relaxation data were acquired to quantify the effect of iron accumulation with parameters describe above. Other parameters for these sequences were FOV = 80 mm × 80 mm, section thickness = 1.0–2.0 mm and no intersection gap, reconstruction matrix = 512 × 512. After in vitro MRI, the cells were suspended in 1 M HCl solution for thorough release and complete dissolution of nanoparticles, then iron concentration was determined by atomic absorption spectrometry (ASS, Z-200; Hitachi, Tokyo, Japan). All experiments were conducted in triplicate.

### 4.5. Intracellular Distribution of Nanoparticles

To assess the intracellular distribution of ASP-SPIONs, 1 × 10^6^ cells were labeled under the optimal conditions (at iron concentration of 30 μg/mL and 1 h of incubation). Afterwards, the cells were washed twice with PBS, and were detected by Prussian blue staining and TEM. For Prussian blue staining, the cells were incubated with the solution containing 10% hydrochloride and 10% potassium ferrocyanide (II) trihydrate for 30 min at 37 °C, followed by nuclear fast red. For TEM, MSCs were fixed in 2.5% glutaraldehyde-cacodylate buffer at 4 °C overnight and postfixed in 1% osmium tetraoxide for 1 h. The cells were then dehydrated in a graded ethanol series and embedded in artificial resin (Epon; Merck, Darmstadt, Germany). Ultrathin sections were made and stained with uranyl acetate and lead citrate, then imaged with an H-7650 transmission electron microscope (Hitachi, Tokyo, Japan) at 80–120 kV. Unlabeled cells served as controls.

### 4.6. Labeling Safety

Labeling safety, cell viability, apoptosis, intracellular ROS level, mitochondrial transmembrane potential and multilineage differentiation capacity were determined by using the CCK-8 assay, Annexin V/propidium iodide (PI) double staining method, ROS assay, and the dual-emission potential sensitive probe 5,5′,6,6′-tetrachloro-1,1′,3,3′-tetraethyl-imidacarbocyanine iodide (JC-1), respectively. The differentiation ability of labeled cells into osteocytes, adipocytes and chondrocytes was also detected.

Cell proliferation and viability were evaluated 0, at 24 h and 48 h after initial labeling under the pre-determined optimal conditions using the CCK-8 assay (Dojindo, Kumamoto, Japan), following the manufacturer’s protocols. Briefly, cells were planted in flat-bottom 96-well plates at a density of 5 × 10^3^ cells per well, with the total culture medium of 200 µL. Then, 10 µL of CCK-8 reagent was added into each well and incubated with cells for 2 h. After that, the absorbance of the formazan product was recorded at 450 nm on a microplate reader (SpectraMaxM5; Molecular Devices, Sunnyvale, CA, USA). Unlabeled cells in the 6 wells were used as controls. Cell apoptosis was assessed by Annexin V-APC/Propidium iodide (PI) double staining method and analyzed using a flow cytometry 0, at 24 and 48 h after labeling. Briefly, 5 × 10^5^ labeled and unlabeled cells were collected and washed twice with ice-cold PBS. They were then resuspended in 200 µL of Annexin-binding buffer solution and stained in the dark with 10 µL Annexin V-APC (marker for apoptosis) and 5 µL PI (marker for necrosis) for 15 min. Cells were analyzed using a flow cytometry (FACScalibur, Becton Dickinson, Mountain View, CA, USA), and apoptosis rate was measured. To determine the intracellular ROS level, ROS assay kit (Genmed, Shanghai, China) was used to assess ROS level 0, at 24 and 48 h after labeling, according to the manufacturer’s manual. Briefly, cells were collected and washed with PBS, and incubated with CM-H_2_DCFDA, which is a non-fluorescent agent that emits fluorescence when the ROS level inside cells increases. After incubation at 37 °C for 20 min, the samples were then immediately analyzed by flow cytometry (FACScalibur, Becton Dickinson, Mountain View, CA, USA) to determine the intensity of fluorescence in the labeled and unlabeled cells. To investigate the early changes that occurred in the mitochondrial transmembrane potential, JC-1 was applied. Briefly, the cells were loaded with 1 × JC-1, which is a green-fluorescent probe that forms red-fluorescent aggregates with an elevation of the mitochondrial membrane potential. After incubation at 37 °C for 20 min, the cells were washed and analyzed by a flow cytometry. The experiments were completed in triplicate.

To evaluate the effect of ASP-SPIONs on the differentiation capability of MSCs, 1 × 10^6^ labeled and unlabeled cells were cultured in osteogenic, adipogenic, and chondrogenic induction media for 2 or 3 weeks (Cyagen, Guangzhou, China), as previously described [47]. Then, the cells were stained with Alizarin red dye staining (detection of calcium deposits) for osteogenic differentiation, oil red O dye staining (detection of lipid) for adipogenic differentiation, and alcian blue staining (detection of mucopolysaccharide) for chondrogenic differentiation, respectively.

### 4.7. Animal Surgery and Cell Transplantation

A left cerebral acute ischaemic infarction was established by transient (2 h) intraluminal suture occlusion of the middle cerebral artery (MCAO) in 18 adult rats, as described previously [45]. Two days after surgery, animals were randomized into three groups (*n* = 6 in each group) to receive a stereotactic injection of 5 × 10^5^ MSCs pre-labeled with ASP-SPIONs under the pre-determined optimal conditions (ASP-SPIONs group), 5 × 10^5^ unlabeled MSCs (unlabeled group), or same volume of PBS (PBS group). The cells were injected into the left striatum (stereotaxic coordinates: 3.0 mm lateral to bregma, 0.5 mm rostral to bregma and 6.0 mm deep from the pial surface) via a 25-µL Hamilton syringe. Before injection, cell viability was determined to be greater than 90%. The cell suspension in PBS was injected at a constant rate of 0.2 µL/min. After injection, the needle was kept in place for another 15 min, and then slowly removed from the brain.

### 4.8. In Vivo MRI

To detect the distribution and migration of the implanted MSCs, in vivo MRI was performed on a clinical 3.0-T system (Intera; Philips Medical Systems, Best, The Netherlands) by using a 50 mm × 50 mm 4-channel phased array rat coil (Suzhou Medcoil Healthcare Co., Ltd, Suzhou, China) at 1, 2, 3, 4, 5, and 6 weeks after transplantation. Coronal MRI images were obtained by using FSE T2WI (TR/TE = 800/60 ms; NSA = 2), proton-density-weighted (PDW) imaging (TR/TE = 3000/20 ms; NSA = 3) and FFE T2*WI (TR/TE = 500/18 ms; Flip angel = 20°; NSA = 3). Other parameters for these sequences were field of view (FOV) = 60 mm, matrix = 256 × 256, section thickness/gap = 1.0/0.0 mm. 

### 4.9. Therapeutic Effects

To evaluate the therapeutic effects of MSCs on stroke, the cerebral infarct volume was measured on in vivo MRI, and functional recovery was assessed by behavioral tests. In brief, the infarct volume was measured on T2WI with ImageJ software (National Institutes of Health). For each slice, the hyperintense (bright) cerebral infarct region and contralateral hemisphere area were manually outlined in a blinded manner. Slice hyperintense areas were then summated and converted to a volume of the infarction as a percentage of contralateral intact brain volume [48]. For behavioral tests, modified neurological severity scores (mNSS) were used to evaluate sensorimotor deficits, as previously described [49]. Neurological function was graded on a scale of 0 to 18 (normal score, 0; maximal deficit score, 18). 

### 4.10. Histology

At 6 weeks after transplantation after MRI, animals were sacrificed and brains were cryosectioned into contiguous coronal 12-µm thickness sections for Prussian blue staining and immunostaining for neuronal nuclear antigen (NeuN, specific for neurons), glial fibrillary acidic protein (GFAP, specific for astrocytes), and CD11b (specific for macrophages/microglias), as previously described [45]. For Prussian blue staining, sections were counterstained with nuclear fast red after being immersed for 30 min in working fluid. For fluorescence immunostaining, the slices were blocked by goat serum for 30 min at room temperature, then incubated with primary antibodies against NeuN (1:200, Abcam), GFAP (1:200, Abcam, Cambridge, UK), and CD11b (1:200, Abcam), at 4 °C overnight. After being washed with PBS, sections were further incubated with Alexa Fluor 594-conjugated secondary antibodies (1:200, Invitrogen, Carlsbad, CA, USA), at 37 °C in the dark for 1 h. Then, the sections were washed followed by nuclei stained with DAPI for 8 min. After washing with PBS, immunoreactive signals were observed using a confocal microscope (LSM 710; Carl Zeiss, Göttingen, Germany). Transplanted cells were detectable, owing to the green fluorescent label under confocal microscopy.

### 4.11. Statistical Analysis

All data are expressed as mean ± SD, unless stated otherwise. Cell viability data, apoptosis rates, and infarct volumes, are expressed as average rates. Comparisons of T2 value, iron concentration, cell viability, apoptosis, intracellular ROS level, and ratio of the red/green fluorescence intensity between labeled cells and unlabeled control cells were performed by using two independent samples *t*-test. The measured infarct volumes and mNSS were compared among those animals that were transplanted with cells pre-labeled with ASP-SPIONs, with unlabeled cells, and with PBS. This was completed using a repeated-measures one-way analysis of variance, followed by the Student-Newman-Keuls test for comparisons among different time points. Statistical analysis was performed by using SPSS 15.0 software for Windows (Chicago, IL, USA). A value of *p* < 0.05 was considered statistically significant.

## 5. Conclusions

Our results suggested that cationic ASP-SPIONs offers a rapid and highly efficient approach for the magnetic labeling of MSCs, enabling in vivo MRI tracking of transplanted cells in stroke. The ASP-SPIONs have no detrimental effects on cell viability, proliferation, apoptosis, intracellular ROS level, mitochondrial transmembrane potential, and differentiation in vitro. The grafted, labeled cells can retain the magnetic label and remain viable up to 6 weeks in the cerebral ischaemic stroke. Using ASP nanoparticles to label stem cells has a variety of favorable properties, including biocompatible and biodegradable characteristics of biopolymers, good biological safety, a simple and rapid labeling procedure, and has no adverse effects on cell biological behaviors. Moreover, the inherent structure features of ASP enable it to be easily modified with specific ligands, or load a broad array of functional molecules to achieve a theranostic nanoplatform. Cationic SPIONs-loaded ASP nanoparticles offer great potential to be translated into clinical stem cell-based therapies.

## Supplementary Materials

The following are available online at http://www.mdpi.com/2079-4991/7/5/107/s1. Figure S1: FTIR spectra of amylose and ASP, Figure S2: 1H NMR spectra of amylose and ASP, Figure S3: FTIR spectra of amylose, ASP, SPIONs and ASP-SPIONs, Figure S4: TG curves of native SPIONs, ASP and ASP-SPIONs, Figure S5: X-ray diffraction diagrams of native SPIONs and ASP-SPIONs, Figure S6: Magnetization curves of SPIONs and ASP-SPIONs, Figure S7: Aqueous dispersibility of ASP-SPIONs and SPIONs.
