# Supplementary material for: Superparamagnetic Iron Oxide Nanoparticles-Complexed Cationic Amylose for In Vivo Magnetic Resonance Imaging Tracking of Transplanted Stem Cells in Stroke
Source: Nanomaterials (Basel). 2017 May 10;7(5):107. doi: 10.3390/nano7050107 (PMC5449988; doi:10.3390/nano7050107)
Supplement: Supplementary file 1 [file nanomaterials-07-00107-s001.pdf]

Superparamagnetic Iron Oxide Nanoparticles-complexed Cationic Amylose for In Vivo Magnetic Resonance Imaging Tracking of Transplanted Stem Cells in Stroke

Bing-Ling Lin [1,†], Jun-Zhao Zhang [2,†], Lie-Jing Lu [1], Jia-Ji Mao [1], Ming-Hui Cao [1], Xu-Hong Mao [3], Fang Zhang [1], Xiao-Hui Duan [1], Chu-Shan Zheng [1], Li-Ming Zhang [2,3,4,*] and Jun Shen [1,*]

[1] Department of Radiology, Sun Yat-Sen Memorial Hospital, Sun Yat-Sen University, Guangzhou 510120, Guangdong, China; linbling@mail2.sysu.edu.cn (B.L.L.); luliejingsysu@163.com (L.J.L.); canterburybells@126.com (J.J.M.); caominghui1019@163.com (M.H.C.); xinxin110007@126.com (F.Z.); duanxiaohui-128@163.com (X.H.D.); zhengchushan0311@126.com (C.S.Z.)

[2] Department of Polymer and Materials Science, School of Chemistry, Sun Yat-Sen University, Guangzhou 510275, Guangdong, China; zhjunzh3@mail2.sysu.edu.cn

[3] School of Materials Science and Engineering, Sun Yat-Sen University, Guangzhou 510275, Guangdong, China; m13929581729_1@163.com

[4] Key Laboratory for Polymeric Composite and Functional Materials of Ministry of Education, Guangdong Provincial Key Laboratory for High Performance Polymer-based Composites, Key Laboratory of Designed Synthesis and Application of Polymer Material, Sun Yat-Sen University, Guangzhou 510275, Guangdong, China

* Correspondence: ceszhlm@mail.sysu.edu.cn (L.M.Z.); shenjun@mail.sysu.edu.cn (J.S.); Tel.: +86-20-8411-2354 (L.M.Z.); +86-20-8133-2702 (J.S.)

† These authors contribute equally to this work.

Appendix A

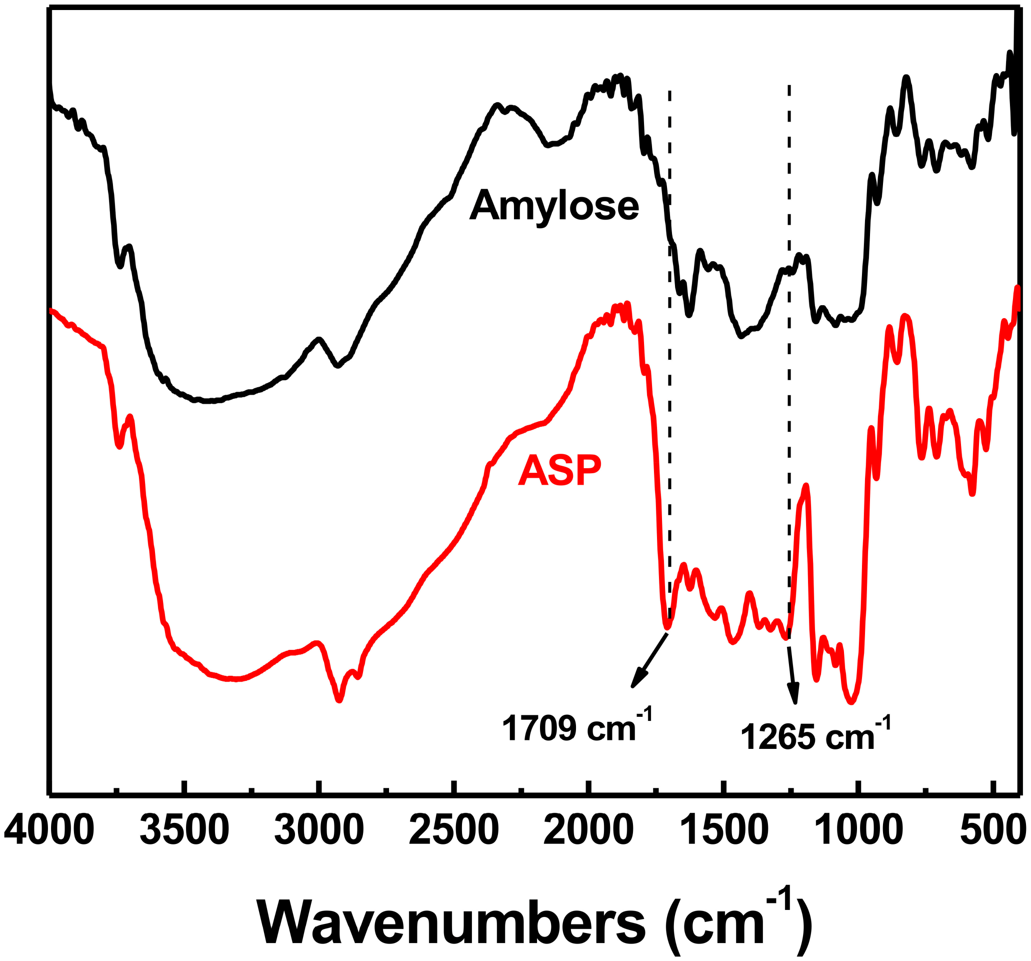

Figure S1. FTIR spectra of amylose and ASP. A new peak at 1709 cm^{-1} and 1265 cm^{-1} of ASP, which could be assigned to the C=O vibration of carbamate structures and C–N vibrations of aliphatic amines, indicate that spermine was grafted to amylose backbone with carbamate linkages.

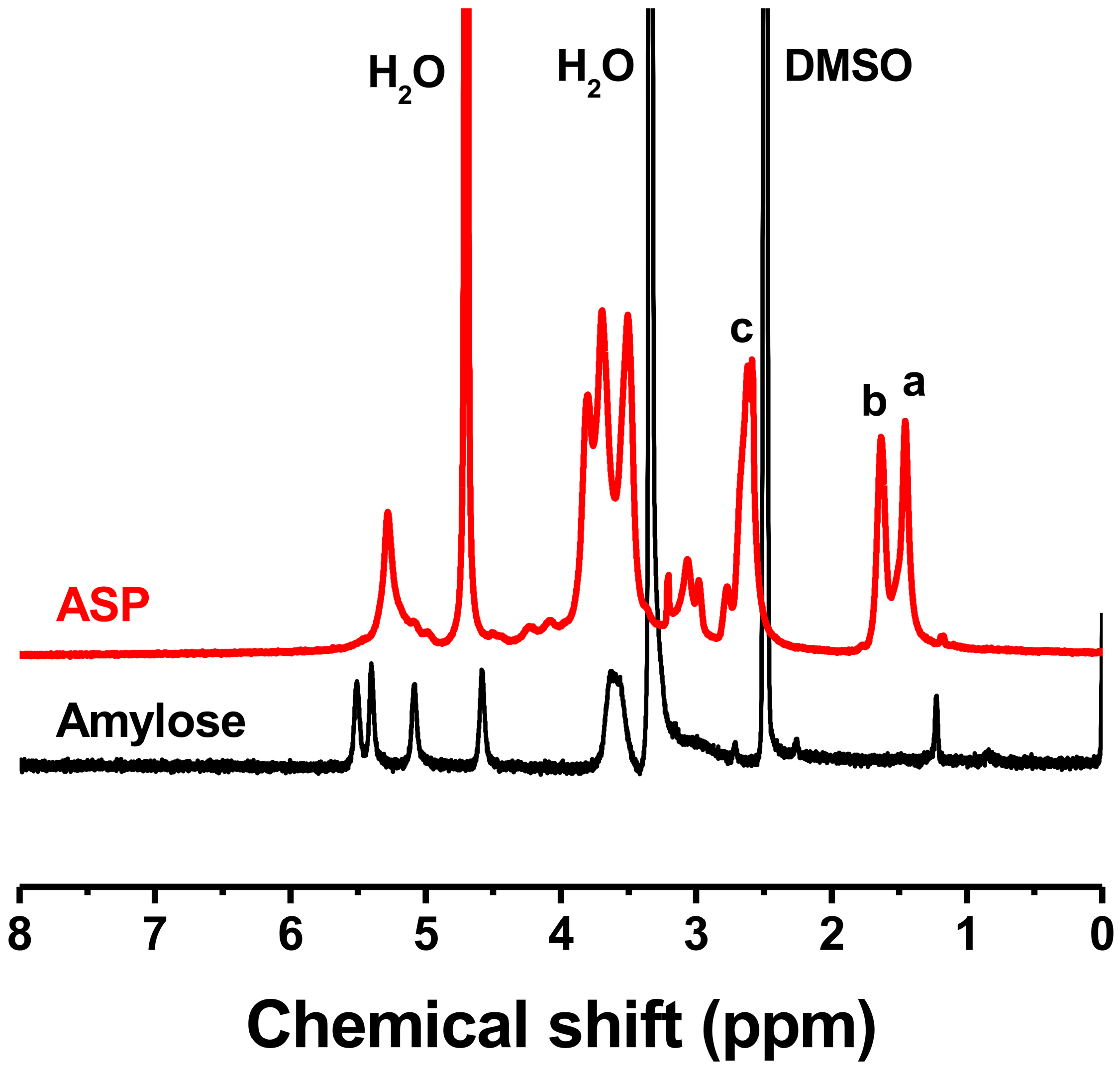

Figure S2. ^{1}H NMR spectra of amylose and ASP. The proton peaks at 1.45, 1.63 and 2.62 ppm further confirmed that the oligoamine residues were conjugated with amylose.

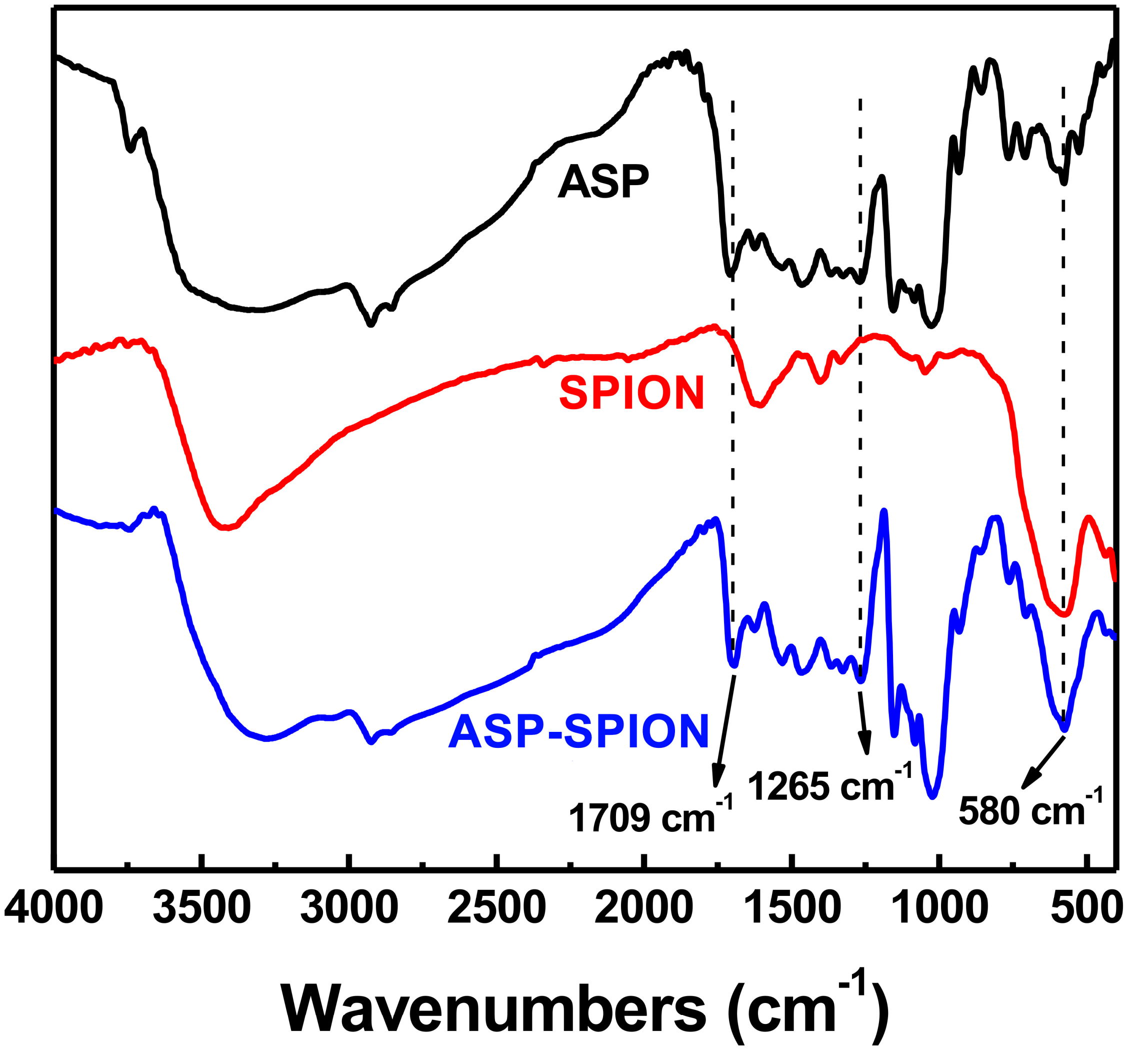

Figure S3. FTIR spectra of amylose, ASP, SPIONs and ASP-SPIONs. Characteristic absorption bands for C=O at 1709 cm^{-1}, C–N at 1265 cm^{-1} and Fe-O at 580 cm^{-1} were observed in ASP-SPION.

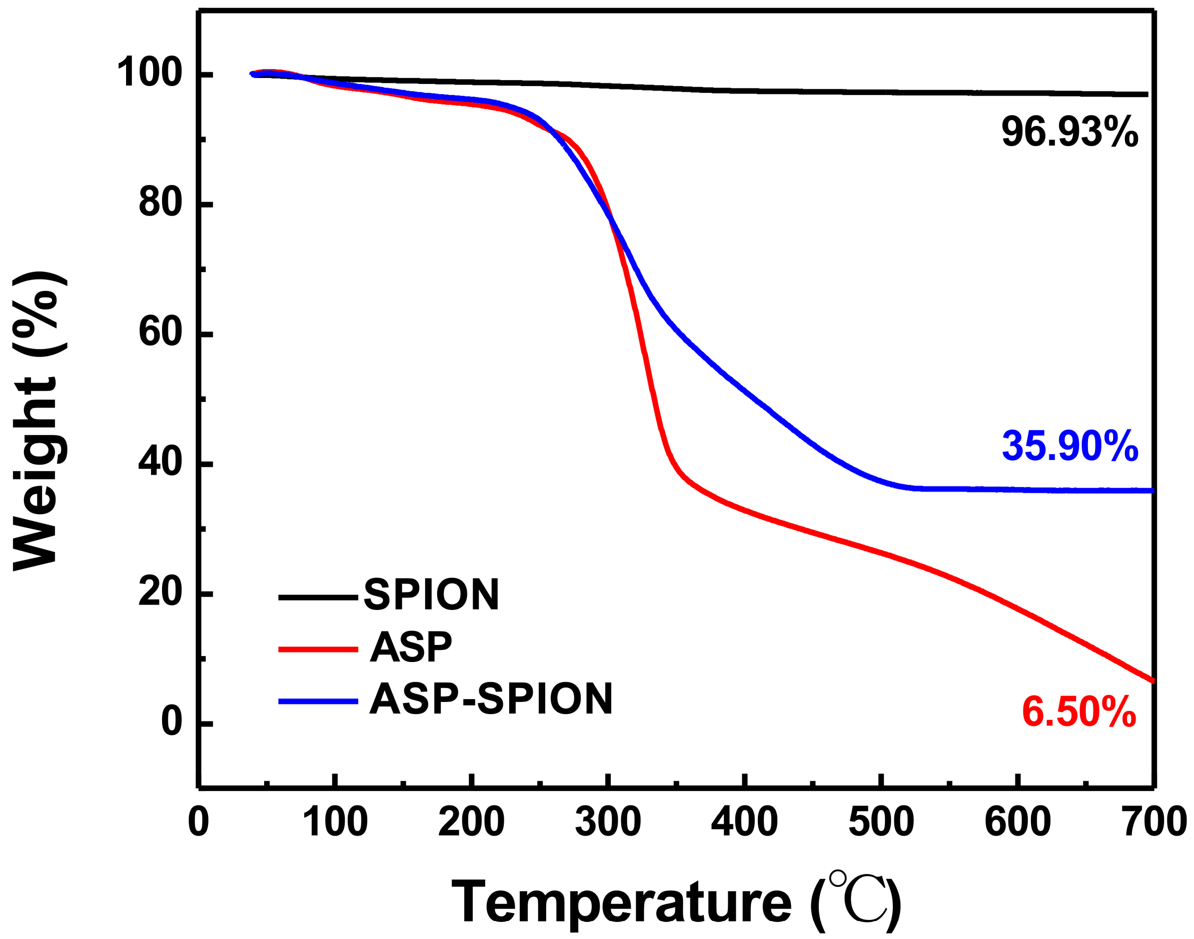

Figure S4. TG curves of native SPIONs, ASP and ASP-SPIONs.

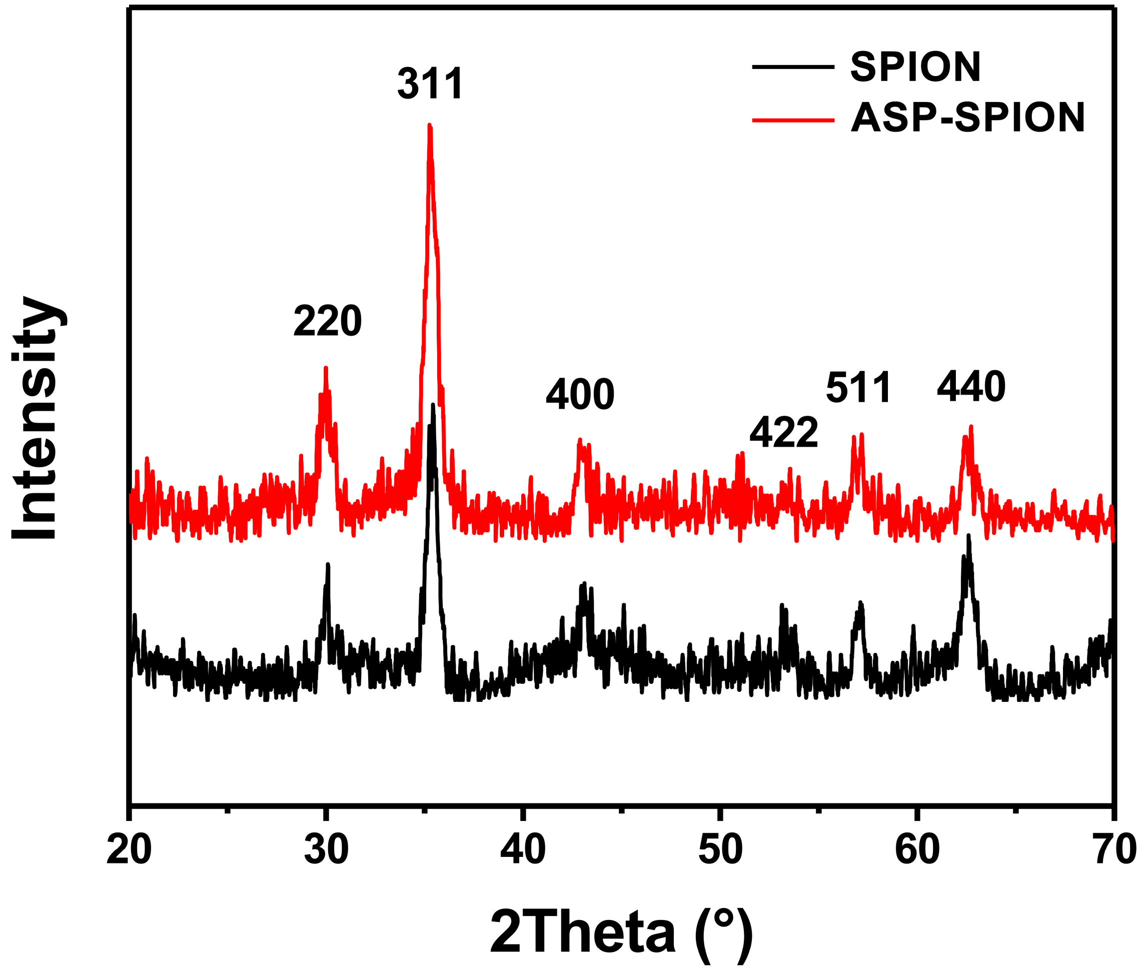

Figure S5. X-ray diffraction diagrams of native SPIONs and ASP-SPIONs.

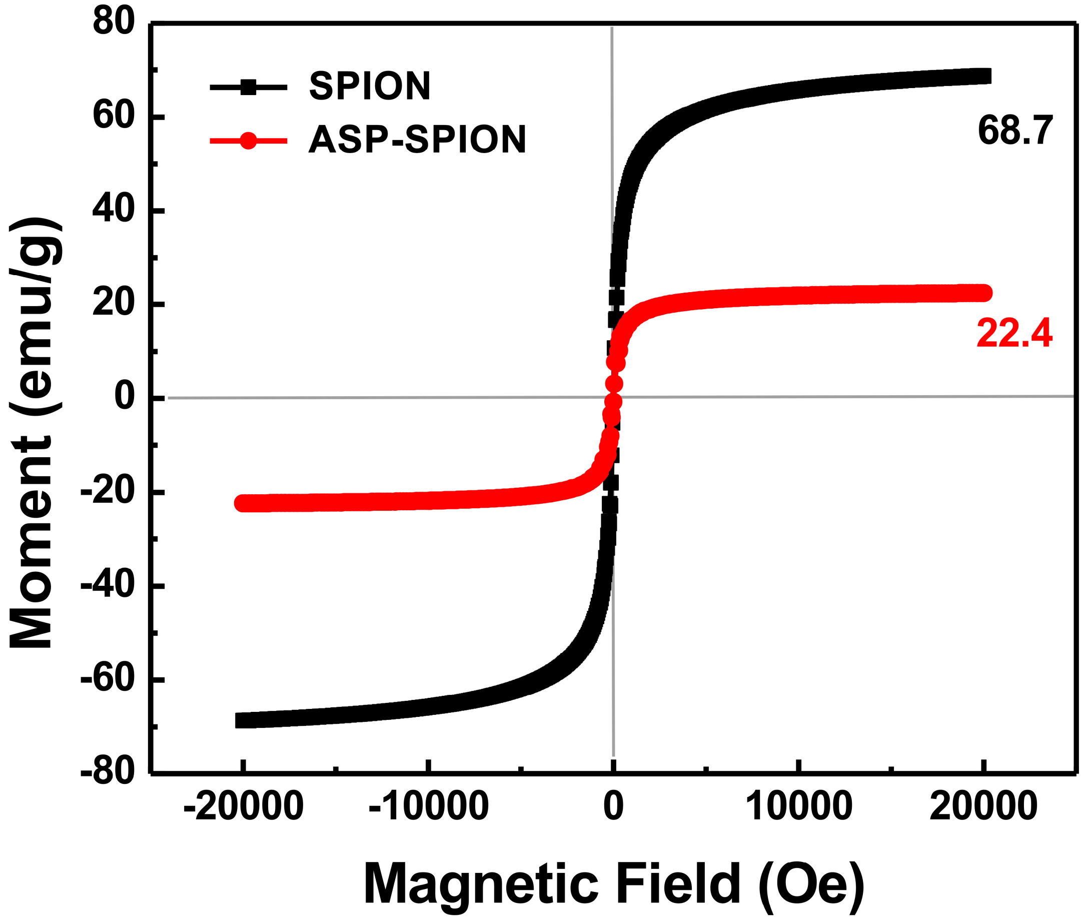

Figure S6. Magnetization curves of SPIONs and ASP-SPIONs. Graph shows that the modification of SPIONs by ASP didn't obviously affect the superparamagnetic property.

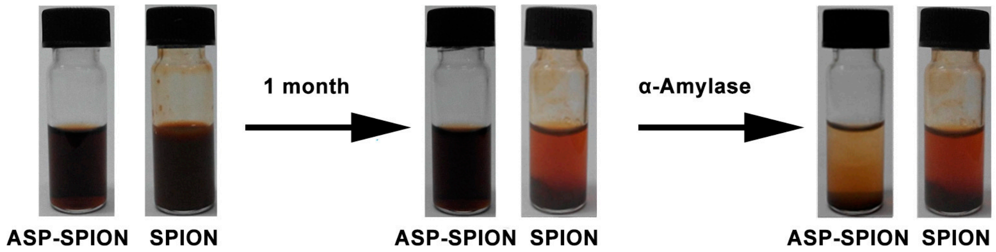

Figure S7. Aqueous dispersibility of ASP-SPIONs and SPIONs.
